# Role of IL-17A rs2275913 and IL-17F rs763780 polymorphisms in risk of cancer development: an updated meta-analysis

**DOI:** 10.1038/srep20439

**Published:** 2016-02-04

**Authors:** Zhi-Ming Dai, Tian-Song Zhang, Shuai Lin, Wang-Gang Zhang, Jie Liu, Xing-Mei Cao, Hong-Bao Li, Meng Wang, Xing-Han Liu, Kang Liu, Shan-Li Li, Zhi-Jun Dai

**Affiliations:** 1 Department of Anesthesiology, The Second Affiliated Hospital of Xi’an Jiaotong University, Xi’an 710004, China; 2 Department of Hematology, The Second Affiliated Hospital of Xi’an Jiaotong University, Xi’an 710004, China; 3 Department Of TCM, The Jing’an District Center Hospital of Shanghai, Shanghai 200040, China; 4 Department of Oncology, The Second Affiliated Hospital of Xi’an Jiaotong University, Xi’an 710004, China; 5 Department of Physiology and Pathophysiology, Xi’an Jiaotong University School of Basic Medical Sciences, Xi’an Jiaotong University Cardiovascular Research Center, Xi’an Jiaotong University Health Science Center, Xi’an 710061, China

## Abstract

Single nucleotide polymorphisms (SNPs) in the interleukin-17 (IL-17) gene have been shown to be correlated with susceptibility to cancer. However, various studies report different results of this association. The aim of the present work was to clarify the effects of IL-17A G197A (rs2275913) and IL-17F T7488C (rs763780) polymorphisms on cancer risk. We performed systematic searches of the PubMed and CNKI databases to obtain relevant publications. Odds ratios (ORs) with 95% confidence intervals (CIs) were used to evaluate the association of rs2275913 and rs763780 polymorphisms with cancer risk. Data were extracted from the selected studies, and statistical analysis was conducted using the STATA software. Our results indicated that rs2275913 and rs763780 polymorphisms significantly increase cancer risk, especially in gastric cancers. Subgroup analysis suggested the existence of a significant correlation between rs763780 polymorphism and cancer susceptibility in Caucasian populations. This updated meta-analysis confirms that rs2275913 and rs763780 polymorphisms are highly associated with increased risk for multiple forms of cancer.

According to the latest global statistics on cancer, approximately 14.1 million new cancer cases and 8.2 million cancer-related deaths occurred worldwide in 2012^1^. Cancer is currently the leading cause of death worldwide, and it represents a major global health concern. Although the pathogenic factors of cancer remain unknown, complex interactions between an individual’s genetic background and environmental factors have been suggested to be highly associated with cancer development^2^.

Inherited factors leading to the development of cancer are not clearly understood, but the roles of cytokines in tumour immunity and carcinogenesis have been well established^3^. Th17 cells, which were identified as a new subset of T helper cells^4^, play pivotal roles in both adaptive and innate immunity, by secreting the pro-inflammatory cytokine interleukin (IL)-17^5^. IL-17 has six family members (IL17A-F) that bind to five receptors (IL-17RA-RD and SEF)^6^. Among all IL-17 family members, IL-17A is one of the most important cytokines, and it may play a role in autoimmune diseases, chronic inflammatory diseases, and malignancies^7^,^8^,^9^; IL-17A has been shown to induce the production of inflammatory chemokines and cytokines by macrophages and neutrophils. More recent studies have reported that IL-17F can also induce the expression of various chemokines, cytokines, and adhesion molecules involved in inflammation-related cancer^10^. The rs763780 variant in the IL-17F gene can lead to a His-to-Arg substitution at amino acid position 161, and thus, inhibit the function of wild-type IL-17F. This may contribute to increased risk of several malignant tumors including gastric cancer, colorectal cancer, and breast cancer^11^,^12^,^13^,^14^.

Meta-analysis is a statistical technique that combines results from different individual studies to produce a comprehensive assessment of the major findings with enhanced accuracy^15^. IL-17 polymorphism has been hypothesized to play a role in carcinogenesis, and numerous studies investigating the same have been published in the past few years^11^,^12^,^13^,^14^,^16^,^17^,^18^,^19^,^20^,^21^,^22^,^23^,^24^,^25^,^26^,^27^,^28^,^29^,^30^,^31^. However, these published studies have reported mixed findings. Therefore, to clarify the role of IL-17A rs2275913 and IL-17F rs763780 polymorphisms in cancer risk, we conducted a comprehensive meta-analysis of all eligible case-control studies.

## Results

### Study characteristics

**Figure 1:**
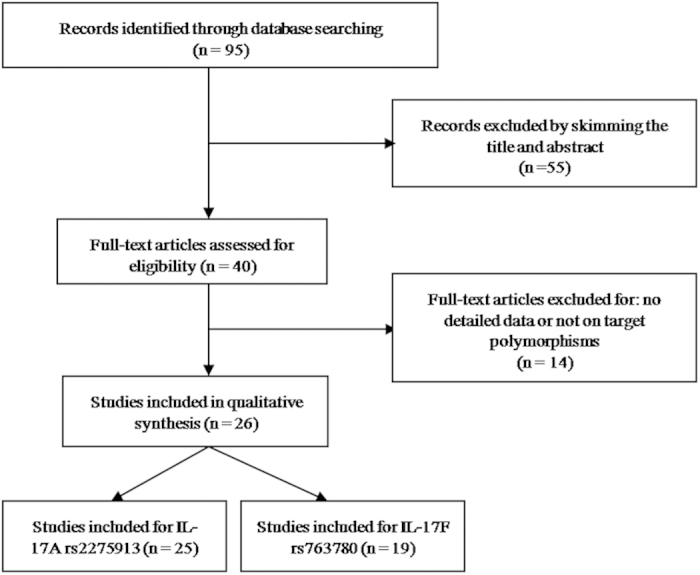
Preferred reporting items for systematic reviews and meta-analyses flow diagram of the literature review process for IL-17 polymorphisms and cancer.

Through primary literature retrieval from Pubmed and CNKI databases, we identified 95 studies that investigated the effect of IL-17 polymorphisms on cancer risk. After screening the titles and abstracts according to the preferred reporting items for systematic reviews and meta-analyses (PRISMA, Fig. 1), 55 studies were excluded from our meta-analysis. Then remaining 40 articles were assessed for eligibility by reading the full-text; 14 articles were excluded owing to either lack of complete data or presence of irrelevant data that focused on other IL-17 polymorphisms. Finally, 26 studies with 7,872 cases and 9,646 cancer-free controls met the inclusion criteria for our meta-analysis for assessing the influence of rs2275913 and rs763780 polymorphisms on cancer risk. Among these, 20 studies were based on the Asian population, and 6 were based on Caucasian populations. The selected studies presented data on several different cancer types: gastric, colorectal, prostate, thyroid, cervical, breast, ovary, bladder, hepatocellular, lung, and oesophageal cancer, and acute myeloid leukaemia. The main characteristics of the included studies are presented in Table 1. The distributions of IL-17A rs2275913 and rs763780 polymorphisms among patients and controls are shown in Table 2.

### Quantitative synthesis results

IL-17A G197A polymorphism (rs2275913).

**Figure 2:**
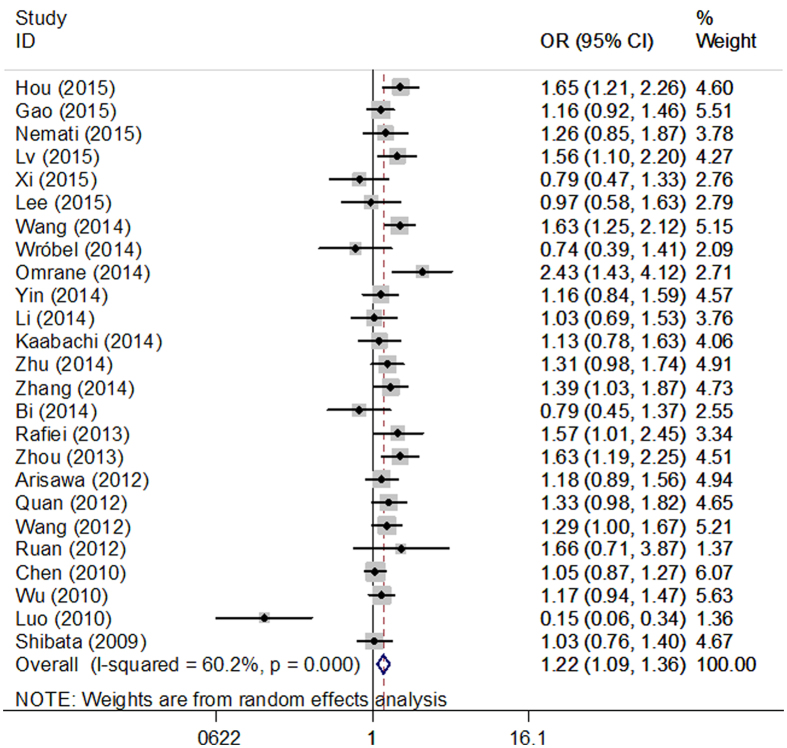
Forest plots of IL-17A rs2275913 polymorphism and cancer risk using a recessive genetic model (AA+AG vs. GG).

Overall, our meta-analysis found a borderline association between rs2275913 polymorphism and increased cancer risk in all genetic models (AA vs. GG: OR = 1.48, 95% CI = 1.25–1.74; AA vs. GG + GA: OR = 1.40, 95% CI = 1.19–1.65; AA + AG vs. GG: OR = 1. 22, 95% CI = 1.09–1.36, Fig. 2; GA vs. GG: OR = 1.13, 95% CI = 1.01–1.28; A vs. G: OR = 1.22, 95% CI = 1.13–1.32) for all cancer types. When only studies following the Hardy–Weinberg equilibrium (HWE) were included in the analysis, a significant association was also observed under all genetic models, and these results are shown in Table 3.

**Figure 3:**
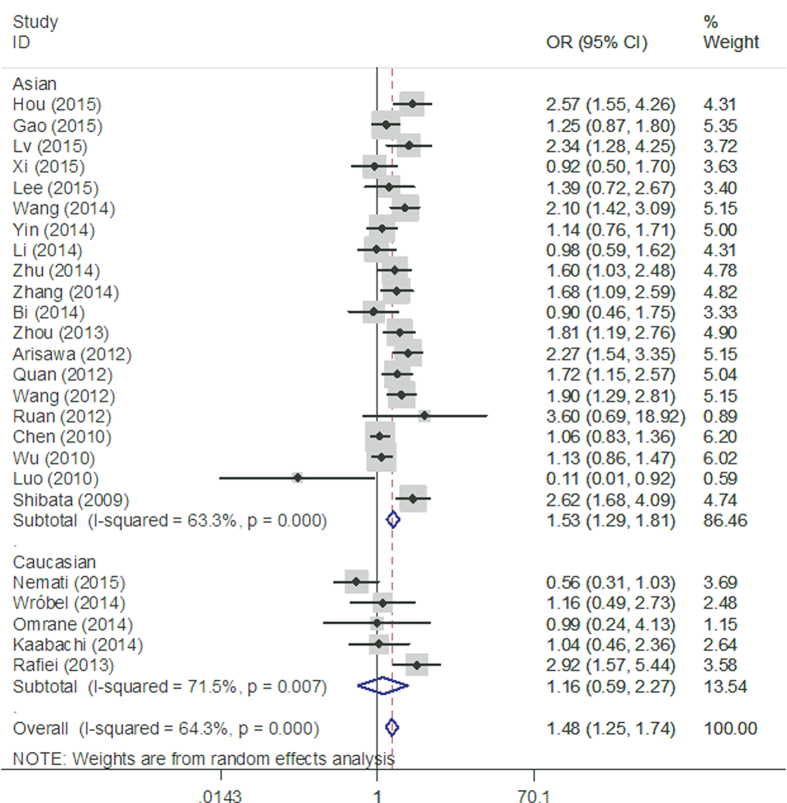
Stratified analysis based on ethnicity for the association between IL-17A rs2275913 polymorphism and cancer risk using a homozygote genetic model (AA vs. GG).

**Figure 4:**
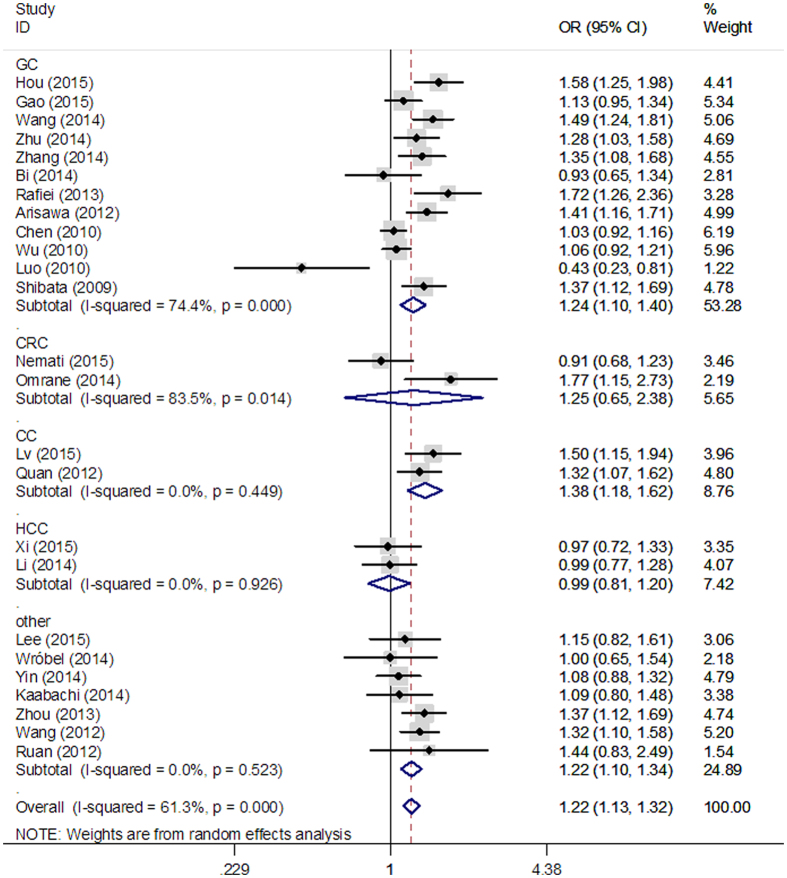
Stratified analysis based on the different cancer sites for the association between IL-17A rs2275913 polymorphism and cancer risk using an allele comparison model (A vs. G).

When subgroup analysis was performed based on ethnicity, no significant correlation was observed between rs2275913 polymorphism and cancer risk in Caucasians. However, statistically significant associations were found in the following genetic models in Asians: (AA vs. GG: OR = 1.53, 95% CI = 1.29–1.81 Fig. 3; AA vs. GG + GA: OR = 1.45, 95% CI = 1.24–1.70; AA + AG vs. GG: OR = 1. 20, 95% CI = 1.06–1.35; A vs. G: OR = 1.22, 95% CI = 1.12–1.32). When results were stratified by cancer type, we found a significant association between rs2275913 polymorphism and increased gastric cancer risk in three genetic models (AA vs. GG: OR = 1.62, 95% CI = 1.26–2.07; AA + AG vs. GG: OR = 1.56, 95% CI = 1.23–1.99; A vs. G: OR = 1.24, 95% CI = 1.10–1.40, Fig. 4). Moreover, significant associations were observed between rs2275913 polymorphism and cervical cancer in all 5 comparison models. All comparisons are listed in Table 3.

IL-17F T7488C polymorphism (rs763780).

**Figure 5:**
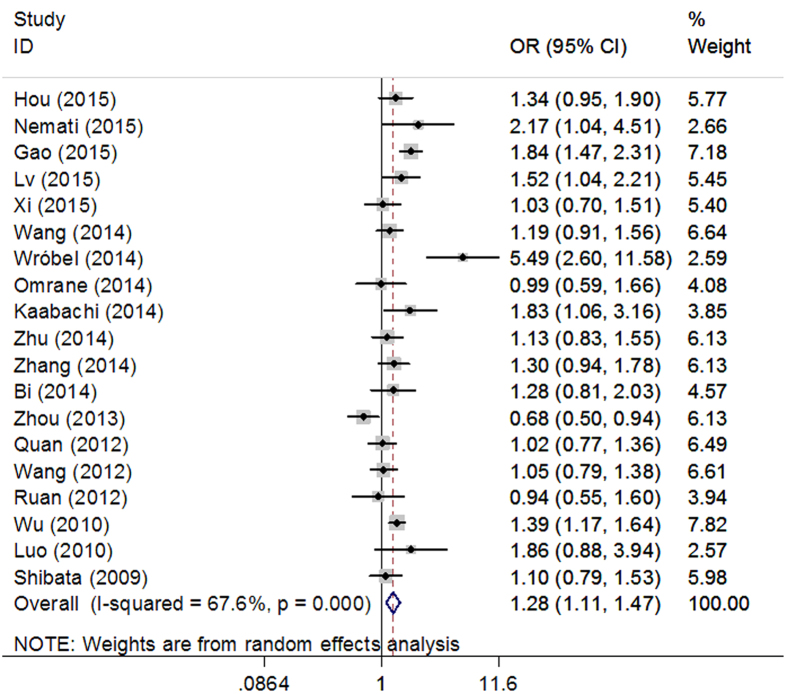
Forest plots of IL-17F rs763780 polymorphism and cancer risk using an allele comparison model (C vs. T).

A significant association was found between rs763780 and cancer susceptibility in 3 different comparison models (CC vs. TT: OR = 1.69, 95% CI = 1.40–2.04; CC vs. TT + TC: OR = 1.64, 95% CI = 1.36–1.97; C vs. T: OR = 1.28, 95% CI = 1.11–1.47, Fig. 5). The same results were observed when studies with HW disequilibrium in controls were excluded.

As shown in Table 3, when subgroup analyses was performed based on ethnicity, significant correlation was observed between rs763780 polymorphism and increased risk of cancer in both Caucasians and Asians. When results were stratified by cancer type, rs763780 polymorphism was found to be significantly associated with an increased risk for gastric cancer in all genetic models (CC vs. TT: OR = 1.67, 95% CI = 1.35–2.06; CC vs. TT + TC: OR = 1.59, 95% CI = 1.29–1.95; CC + TC vs. TT: OR = 1.37, 95% CI = 1.22–1.53; TC vs. TT: OR = 1.28, 95% CI = 1.13–1.45; C vs. T: OR = 1.37, 95% CI = 1.25–1.51).

### Publication bias

**Figure 6:**
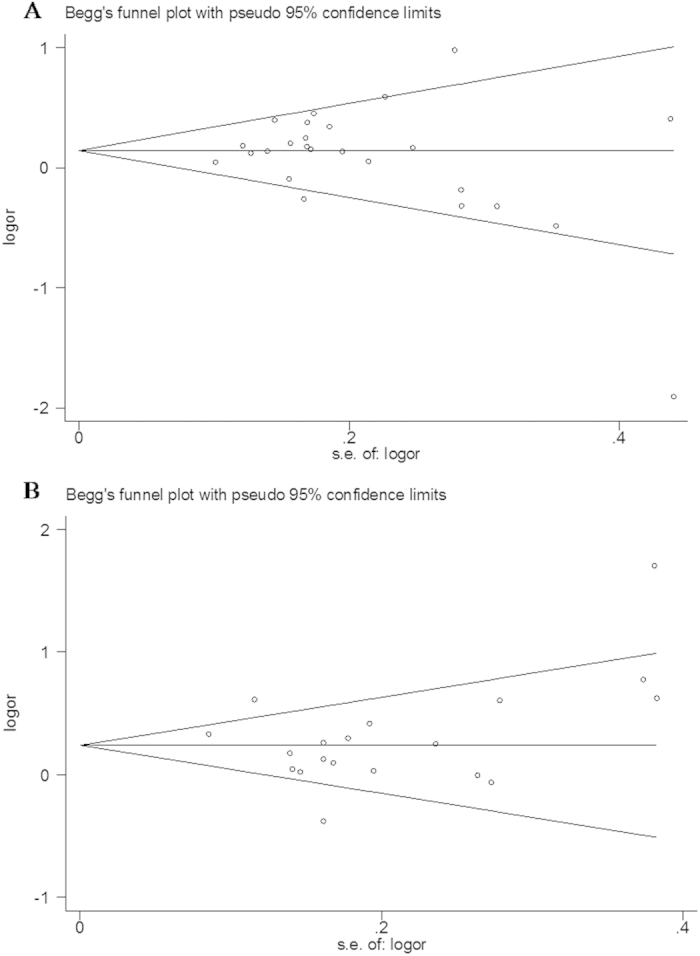
Funnel plot assessing evidence of publication bias from the eligible studies. A. rs2275913; B. rs763780.

Publication bias of the selected articles was assessed using Begg’s funnel plot and Egger’s test. As shown in Fig. 6, the funnel plot was symmetrical in shape, and the P-value of Egger’s test indicated a lack of publication bias for rs2275913 and rs763780 polymorphisms.

### Heterogeneity and sensitivity analyses

**Figure 7:**
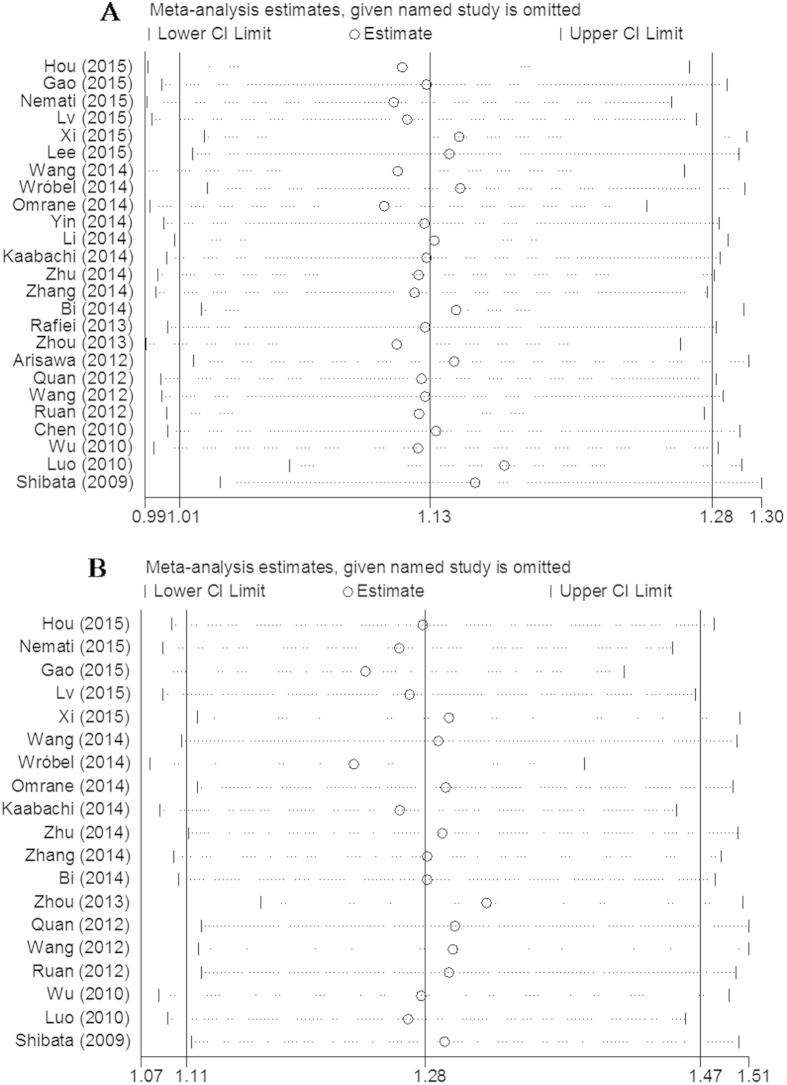
Sensitivity analysis of association between the polymorphisms and cancer risk. A. rs2275913; B. rs763780.

Significant heterogeneities in the data of IL-17A rs2275913 and IL-17F rs763780 polymorphisms were observed in the overall meta-analysis as well as subgroup analysis (Table 4). Due to significant heterogeneity across studies, individual studies used in the meta-analysis were sequentially omitted to to identify the source by sensitivity analysis. The results showed that no individual study skewed the pooled OR values for rs2275913 and rs763780 polymorphisms (Fig. 7).

### Re-sampling statistics

To obtain robust and replicable results, we performed the correlation analysis 10000 times using non-parametric bootstrap re-sampling method. As showed in Supplementary Table S1 & S2, the results indicated that rs2275913 and rs763780 polymorphism in IL-17 gene were consistently associated with cancer risk in different genetic models (P < 0.05).

## Discussion

Recently, inflammatory factors have been shown to increase the risk of developing malignant tumors. IL-17 is a key pro-inflammatory cytokine originally produced by CD4+ memory T cells, and it is involved in both innate and acquired immune responses^32^,^33^. Studies indicate that IL-17 is activated by microbial products, and may promote tumor growth and progression via angiogenic functions^34^,^35^. Aberrant levels of IL-17 have been observed in gastric, colorectal, hepatocellular, ovarian, and breast cancers^36^,^37^,^38^,^39^,^40^.

The IL-17A rs22759133 polymorphism is located in close proximity to 2 nuclear factors activated T cell binding motifs, and it promotes production of high levels of IL-17, which in turn upregulates IL-17-mediated immune responses^41^. IL-17F, another important member of the IL-17 family, plays a key role in neutrophil recruitment and activation by inducing the secretion of cytokines and chemokines. IL-17F rs763780 polymorphism may inhibit the biological activity of IL-17F, and thus contribute to variations in host’s susceptibility to tumors. Data indicate that IL-17A and IL-17F gene polymorphism may play important roles in the pathogenesis of cancer^11^,^13^,^16^,^18^,^24^.

Our study indicated that the two variants of human IL-17 gene significantly increased the risk of cancer in the overall population. When we eliminated studies that deviated from the HWE, similar results were observed. Furthermore, subgroup analyses indicated that associations between these two polymorphisms and cancer risk were also ethnicity- and site-specific. According to the results, rs2275913 polymorphism was significantly associated with elevated cancer risk in Asians (mainly Chinese), but not Caucasians. When subgroup analysis was performed based on cancer types, a significant association was found between rs2275913 polymorphism and risk of gastric/cervical cancer. Interestingly, individuals with the rs2275913 AA genotype showed decreased risk of colorectal cancer as compared to individuals with the GG or GA genotypes. However, only two eligible studies examined IL-17 polymorphisms in colorectal cancer, and therefore, the results may need to be further confirmed. Interestingly, a significant association was found between the rs763780 variant and cancer risk in both the Asian and Caucasian populations. This meta-analysis is, to our knowledge, the first study showing that rs763780 polymorphism increases cancer risk in the Caucasian population.

A meta-analysis by Niu *et al.*^42^ suggested that IL-17 polymorphisms increase the risk of cancer, particularly gastric cancer, in Asian (especially Chinese) populations; our findings were partially in line with results from this meta-analysis. Another meta-analysis by Zhao *et al.*^43^ concluded that not rs763780, but rs2275913, polymorphism may contribute to cancer susceptibility in Asian populations. Long *et al.*^44^ found a positive association between the two polymorphisms and the occurrence of gastric cancer in a meta-analysis, which included 7 independent, case-control studies. Other meta-analyses focused on the association between IL-17F rs763780 polymorphism and cancer risk, and the results indicated that the CC allele might increase the risk of cancer, particularly gastric cancer, in Asian populations^45^,^46^. All of these previous meta-analyses included fewer than 10 eligible case-control studies, with few studies examining Caucasian populations. The present meta-analysis includes 25 independent case-control studies with 7,872 cancer cases and 9,646 cancer-free controls. In addition, 6 of the included studies were based on Caucasians, to more comprehensively evaluate the relationship between IL-17 polymorphisms and cancer risk in Caucasian populations^14^,^16^,^24^,^27^,^31^,^47^.

We conducted sensitivity analysis to confirm the validity of the results presented in our meta-analysis, and studies in which the genotype frequencies in the control group deviated from the HWE were excluded. Results showed that no individual study skewed the overall OR value.

Some limitations of the present meta-analysis should be addressed. First, although significant associations were found between the two polymorphisms and the risk of cancer in multiple genetic models, some potential sources of heterogeneity, such as source of controls, lifestyle, and environmental exposures, were not explored. In addition, some cancer types included in this meta-analysis were investigated only in 1 or 2 studies (Supplementary Table S3), which led to heterogeneity in quantitative analysis. Second, the study results included in this meta-analysis were based on unadjusted analyses, and therefore, we could not estimate the risk of cancer with respect to environmental factors, age, family history, lifestyle, and other risk factors that might have influenced the pooled results. Third, we did not include any studies on the African population, and therefore, the results should be interpreted with caution when extrapolating them to the overall population. Lastly, the study with relative smaller sample size is more likely to be lack of sufficient statistical power to influence the overall results.

Our study represents a comprehensive meta-analysis of the role of IL-17A rs2275913 and IL-17F rs763780 polymorphisms in cancer risk. The results demonstrated that these two polymorphisms significantly increase the risk of development of cancer, particularly gastric cancer. Further large-scale, multicentre studies are required to confirm the pre-diagnostic effect of IL-17 gene polymorphisms on the risk of cancer.

## Material and Methods

### Identification of eligible studies

Systematic article search and quantitative analysis were performed, and written reports were generated according to the Meta-analysis of Observational Studies in Epidemiology guidelines^48^. Eligible studies with publication dates up to March 2015 were obtained through the Pubmed and Chinese National Knowledge Infrastructure (CNKI) databases. No language or geographical restriction was placed for study selection. The keywords search was performed with or without the Medical Subject Headings (MeSH) terms for: ‘interleukin-17/IL-17’, ‘polymorphism’, and ‘cancer’. Additionally, the references in the retrieved articles were manually screened for potential eligible studies.

### Inclusion and exclusion criteria

Studies included in our meta-analysis were required to meet the following criteria: (1) a case-control design; (2) the study goal was to evaluate the association of IL17A rs2275913 and IL-17F rs763780 polymorphisms with cancer risk; (3) the study offered available information on genotype frequency, (4) the controls used had no malignant disease. The following were used as our exclusion criteria: (1) the study was a repeat studies, reviews, or abstracts; (2) the study design was based on family cancers; (3) the study did not include a control group; (4) the study did not investigate the effect of polymorphism; (5) duplicate data.

### Data extraction

Two authors independently selected the potentially relevant studies for data extraction. We screened the titles and abstracts of the studies that met our inclusion criteria. If the content of the abstract was relevant, full articles were read to extract related information. For each eligible study included in our meta-analysis, we obtained information pertaining to first author, years of publication, country of origin, racial ancestry, cancer types, source of control, genotyping method, total number of cases and controls, and P value of Hardy–Weinberg equilibrium (HWE). All cancers were confirmed by histology or pathology. All the case and control groups were well controlled.

### Resampling

We applied re-sampling statistic to examine the robustness of the associations, and 10000 re-sampling analyses were conducted using the bootstrap re-sampling procedure^49^. All the re-sampling analyses were performed by R 3.2.2 software using non-parametric bootstrapping method. The 95% confidence intervals (95% CIs) were estimated by bias-corrected and accelerated (BCa) and overall odds ratios (ORs) were calculated containing all samples under the five different genetic models.

### Statistical analysis

The strength of association between IL-17A rs2275913 and IL-17F rs763780 polymorphisms and cancer risk was assessed as ORs with corresponding 95% CIs based on the allele frequencies in cases and controls of each study selected. The summary OR was calculated according to Woolf’s method. Five different ORs were calculated: dominant model (BB+AB vs. AA), recessive model (BB vs. AA+AB), homozygote comparison (BB vs. AA), heterozygote comparison (AB vs. AA) and allele comparison (B vs. A), the A represents the major allele, and the B represents the minor allele. The Chi-square-based Q statistic was implemented to assess heterogeneity among the studies^50^. The controls that departures of the HWE were evaluated for each study using chi-square test.

The effect of heterogeneity using I^2^ test statistical and significance was considered at P < 0.10. In case of a significant heterogeneity, the pooled ORs were analyzed using a random-effects model, otherwise a fixed-effects model should be used. To evaluate the ethnicity-specific and control-specific effects, subgroup analyses were conducted by source of controls, cancer types, controls whether satisfied HWE or not, and features of the population such as ethnicity. Additionally, to estimate the possible sources of bias, we considered the Egger’s test and Begg’s funnel plot. All statistical analyses were calculated with the software STATA (Version 11.0; Stata Corp, College Station, TX). *P*-values less than 0.05 were considered statistically significant.

## Additional Information

**How to cite this article**: Dai, Z.-M. *et al.* Role of IL-17A rs2275913 and IL-17F rs763780 polymorphisms in risk of cancer development: an updated meta-analysis. *Sci. Rep.* **6**, 20439; doi: 10.1038/srep20439 (2016).

## Supplementary Material

Supplementary Information

## Figures and Tables

**Table 1:** Characteristics of the studies included in the meta-analysis.

First author	Year	Ethnicity	Tumor type	case	control	Genotyping medthod	Source of control	No. of SNP	cancer risk
Hou	2015	Asian	GC	326	326	MassARRAY	Population	1, 2	No.1 yes
Nemati	2015	Caucasian	CRC	202	203	PCR-RFLP	Hospital	1^*^, 2	yes
Gao	2015	Asian	GC	572	572	PCR–RFLP	Hospital	1, 2^*^	No.2 yes
Lv	2015	Asian	CC	264	264	PCR–RFLP	Population	1, 2^*^	No.1 yes
Lee	2015	Asian	PTC	94	260	TaqMan	Population	1	no risk
Xi	2015	Asian	HCC	155	171	PCR–RFLP	Hospital	1, 2	no risk
Wang	2014	Asian	GC	462	462	PCR–RFLP	Population	1, 2	No.1 yes
Wróbel	2014	Caucasian	AML	62	125	PCR–RFLP	Population	1, 2	No.2 yes
Omrane	2014	Caucasian	CRC	102	139	TaqMan	Population	1	yes
Omrane	2014	Caucasian	CRC	102	139	TaqMan	Population	2	yes
Yin	2014	Asian	EC	380	380	SNPscan	Hospital	1	yes
Li	2014	Asian	HCC	395	174	PCR–RFLP	Hospital	1	yes
Kaabachi	2014	Caucasian	LC	239	258	PCR–RFLP	Population	1, 2	No.2 yes
Zhu	2014	Asian	GC	311	611	MassARRAY	Hospital	1, 2^*^	No.1 yes
Zhang	2014	Asian	GC	260	512	MassARRAY	Hospital	1^*^, 2^*^	yes
Bi	2014	Asian	GC	99	150	PCR-RFLP	Hospital	1, 2	no risk
Rafiei	2013	Caucasian	GC	161	171	PCR–RFLP	Hospital	1	yes
Zhou	2013	Asian	BLC	301	446	TaqMan	Hospital	1, 2	yes
Arisawa	2012	Asian	GC	337	587	PCR-SSCP	Hospital	1	yes
Quan	2012	Asian	CC	311	463	TaqMan	Hospital	1, 2	No.1 yes
Wang	2012	Asian	BC	491	502	SNaPshot	Population	1, 2	No.1 yes
Ruan	2012	Asian	OC	92	38	PCR-RFLP	Hospital	1^*^, 2^*^	no risk
Chen	2010	Asian	GC	1042	1090	TaqMan	Population	1	no risk
Wu	2010	Asian	GC	1010	800	PCR–RFLP	Population	1, 2	No.2 yes
Luo	2010	Asian	GC	24	50	PCR-RFLP	Hospital	1^*^, 2	No.1 yes
Shibata	2009	Asian	GC	287	524	PCR–SSCP	Hospital	1^*^, 2	No.1 yes

^*^The *P*-values of the Hardy-Weinberg equilibrium test of control group less than 0.05.

CRC: colorectal cancer; GC: gastric cancer; CC: cervical cancer; PTC: papillary thyroid cancer; HCC: hepatocellular carcinoma; LC: lung cancer; AML: acute myeloid leukemia; EC: esophageal cancer; BLC: bladder cancer; BC: breast cancer; OC: ovarian cancer; NA: not available; PCR-RFLP: polymerase chain reaction restriction fragment length polymorphism; SSCP: single strand conformation polymorphism; SNP: single-nucleotide polymorphisms; No. of SNP: No.1: rs2275913, No.2: rs763780.

**Table 2:** IL-17 polymorphisms Genotype Distribution and Allele Frequency in Cases and Controls.

First author	Genotype (N,%)								Allele frequency (N, %)				MAF
	Case				Control				Case		Control		
	total	AA	AB	BB	total	AA	AB	BB	A	B	A	B	
rs2275913													
Hou 2015	326	121	149	56	326	161	136	29	391	261	458	194	0.40
Nemati 2015	202	100	82	20	199	110	50	39	282	122	270	128	0.30
Gao 2015	572	239	250	83	573	260	241	72	728	416	761	385	0.36
Lv 2015	264	110	117	37	264	139	105	20	337	191	383	145	0.36
Lee 2015	94	28	42	24	260	76	137	47	98	90	289	231	0.48
Xi 2015	155	38	71	46	171	35	90	46	147	163	160	182	0.53
Wang 2014	462	160	211	91	462	214	190	58	531	393	618	306	0.43
Wróbel 2014	62	23	25	14	125	38	67	20	71	53	143	107	0.43
Omrane 2014	102	48	51	3	139	95	38	6	147	57	228	50	0.28
Yin 2014	364	104	180	80	370	117	174	79	388	340	408	332	0.47
Li 2014	391	110	197	84	174	50	85	39	417	365	185	163	0.47
Kaabachi 2014	239	147	80	12	258	166	79	13	374	104	411	105	0.22
Zhu 2014	293	126	122	45	550	273	216	61	374	212	762	338	0.36
Zhang 2014	260	110	102	48	512	258	187	67	322	198	703	321	0.38
Bi 2014	99	32	39	28	150	41	69	40	103	95	151	149	0.48
Rafiei 2013	161	56	61	44	171	78	72	21	173	149	228	114	0.46
Zhou 2013	301	79	154	68	446	164	204	78	312	290	532	360	0.48
Arisawa 2012	333	112	137	84	583	218	293	72	361	305	729	437	0.46
Quan 2012	311	93	142	76	463	168	215	80	328	294	551	375	0.47
Wang 2012	491	165	234	92	501	198	245	58	564	418	641	361	0.43
Ruan 2012	92	20	60	12	38	12	24	2	100	84	48	28	0.46
Chen 2010	1,042	300	522	220	1,090	325	541	224	1,122	962	1,191	989	0.46
Wu 2010	945	210	485	250	768	193	371	204	905	985	757	779	0.52
Luo 2010	24	11	12	1	530	58	426	46	34	14	542	518	0.29
Shibata 2009	287	94	124	69	523	175	299	49	312	262	649	397	0.46
rs763780													
Hou 2015	326	266	38	22	326	278	33	15	570	82	589	63	0.13
Nemati 2015	200	177	23	0	201	190	11	0	377	23	391	11	0.06
Gao 2015	572	420	67	85	572	472	58	42	907	237	1002	142	0.21
Lv 2015	264	209	35	20	264	223	30	11	453	75	476	52	0.14
Xi 2015	155	100	46	9	171	105	63	3	246	64	273	69	0.21
Wang 2014	462	349	98	15	462	362	90	10	796	128	814	110	0.14
Wróbel 2014	62	42	15	5	125	114	11	0	99	25	239	11	0.20
Omrane 2014	100	72	27	1	137	98	38	1	171	29	234	40	0.15
Kaabachi 2014	239	204	34	1	258	236	22	0	442	36	494	22	0.08
Zhu 2014	293	241	35	17	550	463	58	29	517	69	984	116	0.12
Zhang 2014	260	209	30	21	512	429	53	30	448	72	911	113	0.14
Bi 2014	100	69	22	9	150	108	35	7	160	40	251	49	0.20
Zhou 2013	301	240	57	4	446	317	124	5	537	65	758	134	0.11
Quan 2012	311	222	85	4	463	332	126	5	529	93	790	136	0.15
Wang 2012	491	382	103	6	502	396	99	7	867	115	891	113	0.12
Ruan 2012	92	13	69	10	38	2	34	2	95	89	38	38	0.48
Wu 2010	927	540	332	55	777	527	214	36	1412	442	1268	286	0.24
Luo 2010	24	14	10	0	230	176	51	3	38	10	403	57	0.21
Shibata 2009	280	221	55	4	523	419	100	4	497	63	938	108	0.11

A represents the major allele, B represents the minor allele. MAF: minor allele frequencies.

**Table 3:** Summary of ORs and 95% CI of *IL-17A* rs2275913 and *IL-17F* rs763780 polymorphisms with cancer risk.

Comparisons	B vs A		BB vs AA		BB vs AB + AA		BB + AB vs AA		AB vs AA	
	OR (95%CI)	*P*	OR (95%CI)	*P*	OR (95%CI)	*P*	OR (95%CI)	*P*	OR (95%CI)	*P*
rs2275913										
Overall	1.22 (1.13–1.32)	**<0.001**	1.48 (1.25–1.74)	**<0.001**	1.40 (1.19–1.65)	**<0.001**	1.22 (1.09–1.36)	**0.001**	1.13 (1.01–1.28)	**0.04**
HWE										
Yes	1.23 (1.14–1.33)	**<0.001**	1.49 (1.27–1.75)	**<0.001**	1.39 (1.20–1.61)	**<0.001**	1.26 (1.14–1.38)	**<0.001**	1.17 (1.06–1.30)	**0.002**
Ethnicity										
Asian	1.22 (1.12–1.32)	**<0.001**	1.53 (1.29–1.81)	**<0.001**	1.45 (1.24–1.70)	**<0.001**	1.20 (1.06–1.35)	**0.003**	1.10 (0.97–1.24)	0.13
Caucasian	1.24 (0.94–1.63)	0.13	1.16 (0.59–2.27)	0.66	1.08 (0.51–2.28)	0.84	1.34 (0.97–1.84)	0.07	1.35 (0.90–2.03)	0.14
Cancer type										
GC	1.24 (1.10–1.40)	**<0.001**	1.62 (1.26–2.07)	**<0.001**	1.56 (1.23–1.99)	**<0.001**	1.17 (0.99–1.38)	0.07	1.05 (0.89–1.25)	0.56
CRC	1.25 (0.65–2.38)	0.51	0.61 (0.35–1.07)	0.09	0.48 (0.28–0.82)	**0.007**	1.71 (0.90–3.24)	0.10	2.10 (1.49–2.96)	<0.001
CC	1.38 (1.18–1.62)	**<0.001**	1.89 (1.35–2.64)	**<0.001**	1.66 (1.23–2.24)	**0.001**	1.43 (1.14–1.80)	**0.002**	1.29 (1.01–1.64)	**0.04**
HCC	0.99 (0.818–1.20)	0.89	0.96 (0.65–1.41)	0.82	1.03 (0.75–1.42)	0.85	0.94 (0.68–1.28)	0.68	0.92 (0.66–1.29)	0.63
rs763780										
Overall	1.28 (1.11–1.47)	**0.001**	1.69 (1.40–2.04)	**<0.001**	1.64 (1.36–1.97)	**<0.001**	1.25 (1.07–1.47)	**0.001**	1.17 (1.00–1.37)	0.06
HWE										
Yes	1.25 (1.02–1.52)	**0.03**	1.70 (1.21–2.39)	**0.002**	1.69 (1.20–2.38)	**0.002**	1.21 (0.98–1.50)	0.08	1.15 (0.93–1.41)	0.20
Ethnicity										
Asian	1.06 (0.95–1.19)	**0.28**	1.54 (1.08–2.20)	**0.02**	1.55 (1.09–2.20)	**0.02**	1.04 (0.87–1.24)	0.66	0.99 (0.83–1.19)	0.95
Caucasian	2.08 (0.94–1.63)	**0.03**	6.17 (1.50–30.0)	**0.01**	6.19 (1.36–28.1)	**0.02**	2.02 (1.08–3.76)	**0.03**	1.83 (1.08–3.11)	**0.02**
Cancer type										
GC	1.37 (1.25–1.51)	**<0.001**	1.67 (1.35–2.06)	**<0.001**	1.59 (1.29–1.95)	**<0.001**	1.37 (1.22–1.53)	**<0.001**	1.28 (1.13–1.45)	**<0.001**
CRC	1.40 (0.65–3.00)	0.38	–	–	–	–	1.43 (0.63–3.22)	0.39	1.42 (0.63–3.24)	0.40

A: the major allele; B: the minor allele; CI: confidence interval; OR: odds ratio; GC: Gastric cancer; CRC: colorectal cancer; CC: cervical cancer; HCC: hepatocellular carcinoma.

**Table 4:** Heterogeneity-analysis results.

Comparisons	B vs A			BB vs AA			BB vs AA+AB			BB+AB vs AA			AB vs AA		
	I^2^	*P*	EM	I^2^	*P*	EM	I^2^	*P*	EM	I^2^	*P*	EM	I^2^	*P*	EM
rs2275913															
Overall	61%	<0.001	R	64%	<0.001	R	70%	<0.001	R	60%	<0.001	R	62%	<0.001	R
HWE															
Yes	58%	0.001	R	57%	0.001	R	60%	<0.001	R	43%	0.02	R	39%	0.04	R
Ethnicity															
Asian	62%	<0.001	R	63%	<0.001	R	68%	<0.001	R	62%	<0.001	R	60%	<0.001	R
Caucasian	68%	0.01	R	72%	0.007	R	80%	0.001	R	58%	0.05	R	70%	0.009	R
Cancer type															
GC	74%	<0.001	R	75%	<0.001	R	79%	<0.001	R	73%	<0.001	R	71%	<0.001	R
CRC	84%	0.01	R	0%	0.48	F	0%	0.61	F	74%	0.05	R	14%	0.28	F
CC	0%	0.45	F	0%	0.40	F	0%	0.47	F	0%	0.51	F	0%	0.51	F
HCC	0%	0.93	F	0%	0.88	F	0%	0.56	F	0%	0.43	F	8%	0.30	F
rs763780															
Overall	68%	<0.001	R	0%	0.79	F	0%	0.79	F	64%	<0.001	R	57%	0.001	R
HWE															
Yes	68%	<0.001	R	0%	0.79	F	0%	0.82	F	66%	<0.001	R	61%	0.002	R
Ethnicity															
Asian	37%	0.12	F	0%	0.94	F	0%	0.93	F	43%	0.08	R	44%	0.08	R
Caucasian	78%	0.003	R	17%	0.30	F	5%	0.35	F	72%	0.02	R	59%	0.06	R
Cancer type															
GC	31%	0.17	F	6%	0.79	F	0%	0.72	F	10%	0.36	F	0%	0.48	F
CRC	66%	0.09	R	–	–	–	–	–	–	67%	0.08	R	67%	0.08	R

A: the major allele; B: the minor allele; EM: Effects model; F: fixed effects model; R: random effects model; GC: Gastric cancer; CRC: colorectal cancer; CC: cervical cancer; HCC: hepatocellular carcinoma.

## References

[b1] Ferlay J. *et al.* Cancer incidence and mortality worldwide: sources, methods and major patterns in GLOBOCAN 2012. Int J Cancer 136, E359–386, doi: 10.1002/ijc.29210 (2015). 25220842

[b2] Pharoah P. D., Dunning A. M., Ponder B. A. & Easton D. F. Association studies for finding cancer-susceptibility genetic variants. Nature Reviews Cancer 4, 850–860 (2004). 15516958 10.1038/nrc1476

[b3] Smyth M. J., Cretney E., Kershaw M. H. & Hayakawa Y. Cytokines in cancer immunity and immunotherapy. Immunol Rev 202, 275–293, doi: 10.1111/j.0105-2896.2004.00199.x (2004). 15546400

[b4] Harrington L. E. *et al.* Interleukin 17-producing CD4+ effector T cells develop via a lineage distinct from the T helper type 1 and 2 lineages. Nat Immunol 6, 1123–1132, doi: 10.1038/ni1254 (2005). 16200070

[b5] Iwakura Y., Ishigame H., Saijo S. & Nakae S. Functional specialization of interleukin-17 family members. Immunity 34, 149–162, doi: 10.1016/j.immuni.2011.02.012 (2011). 21349428

[b6] Kawaguchi M., Adachi M., Oda N., Kokubu F. & Huang S. K. IL-17 cytokine family. J Allergy Clin Immunol 114, 1265-1273; quiz 1274, doi: 10.1016/j.jaci.2004.10.019 (2004). 15577820

[b7] Kirkham B. W. *et al.* Synovial membrane cytokine expression is predictive of joint damage progression in rheumatoid arthritis: a two-year prospective study (the DAMAGE study cohort). Arthritis Rheum 54, 1122–1131, doi: 10.1002/art.21749 (2006). 16572447

[b8] Fujino S. *et al.* Increased expression of interleukin 17 in inflammatory bowel disease. Gut 52, 65–70 (2003). 12477762 10.1136/gut.52.1.65 PMC1773503

[b9] Su X. *et al.* Tumor microenvironments direct the recruitment and expansion of human Th17 cells. J Immunol 184, 1630–1641, doi: 10.4049/jimmunol.0902813 (2010). 20026736

[b10] Song X. & Qian Y. IL-17 family cytokines mediated signaling in the pathogenesis of inflammatory diseases. Cell Signal 25, 2335–2347, doi: 10.1016/j.cellsig.2013.07.021 (2013). 23917206

[b11] Wang L. *et al.* Association analysis of IL-17A and IL-17F polymorphisms in Chinese Han women with breast cancer. PLoS One 7, e34400, doi: 10.1371/journal.pone.0034400 (2012). 22461912 PMC3312906

[b12] Wu X. *et al.* Association between polymorphisms in interleukin-17A and interleukin-17F genes and risks of gastric cancer. Int J Cancer 127, 86–92, doi: 10.1002/ijc.25027 (2010). 19904747

[b13] Shibata T., Tahara T., Hirata I. & Arisawa T. Genetic polymorphism of interleukin-17A and -17F genes in gastric carcinogenesis. Hum Immunol 70, 547–551, doi: 10.1016/j.humimm.2009.04.030 (2009). 19414056

[b14] Omrane I. *et al.* Significant association between interleukin-17A polymorphism and colorectal cancer. Tumour Biol 35, 6627–6632, doi: 10.1007/s13277-014-1890-4 (2014). 24699997

[b15] Zintzaras E. & Lau J. Synthesis of genetic association studies for pertinent gene-disease associations requires appropriate methodological and statistical approaches. J Clin Epidemiol 61, 634–645, doi: 10.1016/j.jclinepi.2007.12.011 (2008). 18538260

[b16] Nemati K., Golmoghaddam H., Hosseini S. V., Ghaderi A. & Doroudchi M. Interleukin-17FT7488 allele is associated with a decreased risk of colorectal cancer and tumor progression. Gene 561, 88–94, doi: 10.1016/j.gene.2015.02.014 (2015). 25680555

[b17] Gao Y. W., Xu M., Xu Y., Li D. & Zhou S. Effect of three common IL-17 single nucleotide polymorphisms on the risk of developing gastric cancer. Oncol Lett 9, 1398–1402, doi: 10.3892/ol.2014.2827 (2015). 25663919 PMC4314969

[b18] Lv Q. *et al.* Association between six genetic variants of IL-17A and IL-17F and cervical cancer risk: a case-control study. Tumour Biol, doi: 10.1007/s13277-015-3041-y (2015). 25596084

[b19] Lee Y. C. *et al.* Association between interleukin 17/interleukin 17 receptor gene polymorphisms and papillary thyroid cancer in Korean population. Cytokine 71, 283–288, doi: 10.1016/j.cyto.2014.11.011 (2015). 25484349

[b20] Xi X. E. *et al.* Interleukin-17A and interleukin-17F gene polymorphisms and hepatitis B virus-related hepatocellular carcinoma risk in a Chinese population. Med Oncol 32, 355, doi: 10.1007/s12032-014-0355-3 (2015). 25429834

[b21] Wang N. *et al.* IL-17 gene polymorphism is associated with susceptibility to gastric cancer. Tumour Biol 35, 10025–10030, doi: 10.1007/s13277-014-2255-8 (2014). 25012243

[b22] Yin J. *et al.* Interleukin 17A rs4711998 A>G polymorphism was associated with a decreased risk of esophageal cancer in a Chinese population. Dis Esophagus 27, 87–92, doi: 10.1111/dote.12045 (2014). 23895419

[b23] Li N. *et al.* IL17A gene polymorphisms, serum IL-17A and IgE levels, and hepatocellular carcinoma risk in patients with chronic hepatitis B virus infection. Mol Carcinog 53, 447–457, doi: 10.1002/mc.21992 (2014). 23280722

[b24] Kaabachi W. *et al.* Interleukin-17A and -17F genes polymorphisms in lung cancer. Cytokine 66, 23–29, doi: 10.1016/j.cyto.2013.12.012 (2014). 24548421

[b25] Qinghai Z., Yanying W., Yunfang C., Xukui Z. & Xiaoqiao Z. Effect of interleukin-17A and interleukin-17F gene polymorphisms on the risk of gastric cancer in a Chinese population. Gene 537, 328–332, doi: 10.1016/j.gene.2013.11.007 (2014). 24315816

[b26] Zhang X., Zheng L. & Sun Y. Analysis of the association of interleukin-17 gene polymorphisms with gastric cancer risk and interaction with Helicobacter pylori infection in a Chinese population. Tumour Biol 35, 1575–1580, doi: 10.1007/s13277-013-1217-x (2014). 24218334

[b27] Rafiei A. *et al.* Polymorphism in the interleukin-17A promoter contributes to gastric cancer. World J Gastroenterol 19, 5693–5699, doi: 10.3748/wjg.v19.i34.5693 (2013). 24039363 PMC3769907

[b28] Zhou B. *et al.* Interleukin-17 gene polymorphisms are associated with bladder cancer in a Chinese Han population. Mol Carcinog 52, 871–878, doi: 10.1002/mc.21928 (2013). 22692973

[b29] Arisawa T. *et al.* Genetic polymorphisms of IL17A and pri-microRNA-938, targeting IL17A 3’-UTR, influence susceptibility to gastric cancer. Hum Immunol 73, 747–752, doi: 10.1016/j.humimm.2012.04.011 (2012). 22537748

[b30] Quan Y. *et al.* Association between IL17 polymorphisms and risk of cervical cancer in Chinese women. Clin Dev Immunol 2012, 258293, doi: 10.1155/2012/258293 (2012). 23049595 PMC3463183

[b31] Wrobel T. *et al.* IL-17F gene polymorphism is associated with susceptibility to acute myeloid leukemia. J Cancer Res Clin Oncol 140, 1551–1555, doi: 10.1007/s00432-014-1674-7 (2014). 24793548 PMC4131129

[b32] Korn T., Bettelli E., Oukka M. & Kuchroo V. K. IL-17 and Th17 Cells. Annu Rev Immunol 27, 485–517, doi: 10.1146/annurev.immunol.021908.132710 (2009). 19132915

[b33] Moseley T. A., Haudenschild D. R., Rose L. & Reddi A. H. Interleukin-17 family and IL-17 receptors. Cytokine Growth Factor Rev 14, 155–174 (2003). 12651226 10.1016/s1359-6101(03)00002-9

[b34] Shime H. *et al.* Tumor-secreted lactic acid promotes IL-23/IL-17 proinflammatory pathway. J Immunol 180, 7175–7183 (2008). 18490716 10.4049/jimmunol.180.11.7175

[b35] Kato T. *et al.* Expression of IL-17 mRNA in ovarian cancer. Biochem Biophys Res Commun 282, 735–738, doi: 10.1006/bbrc.2001.4618 (2001). 11401524

[b36] Chen J. G. *et al.* Intratumoral expression of IL-17 and its prognostic role in gastric adenocarcinoma patients. Int J Biol Sci 7, 53–60 (2011). 21234303 10.7150/ijbs.7.53 PMC3020363

[b37] Straus D. S. TNFalpha and IL-17 cooperatively stimulate glucose metabolism and growth factor production in human colorectal cancer cells. Mol Cancer 12, 78, doi: 10.1186/1476-4598-12-78 (2013). 23866118 PMC3725176

[b38] Liao R. *et al.* High expression of IL-17 and IL-17RE associate with poor prognosis of hepatocellular carcinoma. J Exp Clin Cancer Res 32, 3, doi: 10.1186/1756-9966-32-3 (2013). 23305119 PMC3621615

[b39] Lan C. *et al.* High density of IL-17-producing cells is associated with improved prognosis for advanced epithelial ovarian cancer. Cell Tissue Res 352, 351–359, doi: 10.1007/s00441-013-1567-0 (2013). 23397428

[b40] Zhu X. *et al.* IL-17 expression by breast-cancer-associated macrophages: IL-17 promotes invasiveness of breast cancer cell lines. Breast Cancer Res 10, R95, doi: 10.1186/bcr2195 (2008). 19014637 PMC2656888

[b41] Liu X. K., Lin X. & Gaffen S. L. Crucial role for nuclear factor of activated T cells in T cell receptor-mediated regulation of human interleukin-17. J Biol Chem 279, 52762–52771, doi: 10.1074/jbc.M405764200 (2004). 15459204

[b42] Niu Y. M., Yuan H. & Zhou Y. Interleukin-17 gene polymorphisms contribute to cancer risk. Mediators Inflamm 2014, 128490, doi: 10.1155/2014/128490 (2014). 25147431 PMC4131465

[b43] Zhao H. Y., Wang R. & Ma W. IL-17A G197A and IL-17F T7488C polymorphisms and cancer risk in Asian populations: a meta-analysis. J BUON 19, 562–566 (2014). 24965422

[b44] Long Z. W. *et al.* Association of IL-17 polymorphisms with gastric cancer risk in Asian populations. World J Gastroenterol 21, 5707–5718, doi: 10.3748/wjg.v21.i18.5707 (2015). 25987798 PMC4427697

[b45] Yao F. *et al.* Role of IL-17F T7488C polymorphism in carcinogenesis: a meta-analysis. Tumour Biol 35, 9061–9068, doi: 10.1007/s13277-014-2171-y (2014). 24913709

[b46] Chen X. J., Zhou T. Y., Chen M. & Pu D. Meta analysis of association of the IL-17F rs763780T>C gene polymorphism with cancer risk. Asian Pac J Cancer Prev 15, 8083–8087 (2014). 25338988 10.7314/apjcp.2014.15.19.8083

[b47] Omrane I. *et al.* Significant association between IL23R and IL17F polymorphisms and clinical features of colorectal cancer. Immunol Lett 158, 189–194, doi: 10.1016/j.imlet.2014.01.002 (2014). 24440568

[b48] Stroup D. F. *et al.* Meta-analysis of observational studies in epidemiology: a proposal for reporting. Meta-analysis Of Observational Studies in Epidemiology (MOOSE) group. JAMA 283, 2008–2012 (2000). 10789670 10.1001/jama.283.15.2008

[b49] Li J. *et al.* Identification of high-quality cancer prognostic markers and metastasis network modules. Nat Commun 1, 34, doi: 10.1038/ncomms1033 (2010). 20975711 PMC2972666

[b50] Higgins J. P. T. & Thompson S. G. Quantifying heterogeneity in a meta-analysis. Statistics in Medicine 21, 1539–1558, doi: Doi 10.1002/Sim.1186 (2002). 12111919

