# Supplementary material for: Role of IL-17A rs2275913 and IL-17F rs763780 polymorphisms in risk of cancer development: an updated meta-analysis
Source: Sci Rep. 2016 Feb 4;6:20439. doi: 10.1038/srep20439 (PMC4740815; doi:10.1038/srep20439)
Supplement: Supplementary Information [file srep20439-s1.pdf]

Role of IL-17A rs2275913 and IL-17F rs763780 polymorphisms in risk of cancer development: an updated meta-analysis

Zhi-Ming Dai, Tian-Song Zhang, Shuai Lin, Wang-Gang Zhang, Jie Liu, Xing-Mei Cao, Hong-Bao Li, Meng Wang, Xing-Han Liu, Kang Liu, Shan-Li Li, Zhi-Jun Dai

Supplementary Table S1. Re-sampling analysis of the associations between IL-17 rs2275913 polymorphism and cancer risk

gene model	OR (95% CI)	*P*
dominant model	1.24 (1.15-1.37)	<0.0001
recessive model	1.40 (1.21-1.62)	<0.0001
homozygote comparison	1.48 (1.28-1.73)	<0.0001
heterozygote comparison	1.16 (1.07-1.28)	0.0008
allelic model	1.23 (1.14-1.32)	<0.0001

Supplementary Table S2. Re-sampling analysis of the associations between IL-17 rs763780 polymorphism and cancer risk

gene model	OR (95% CI)	*P*
dominant model	1.32 (1.12-1.70)	0.003
recessive model	1.61 (1.36-1.97)	<0.0001
homozygote comparison*	-	-
heterozygote comparison	1.21 (1.04-1.46)	0.02
allelic model	1.36 (1.17-1.84)	0.0007

*Data does not apply to the model, and the relevant statistics cannot be calculated without reporting

Supplementary Table S3. The associations between IL-17 polymorphisms and other cancers included in the meta-analysis

Cancer type	No. of studies	Author	Year	Ethnicity	Case/control	Polymorphism site	Cancer risk
CRC	2	Nemati	2015	Caucasian	202/203	rs2275913, rs763780	yes
		Omrane	2014	Caucasian	102/139	rs2275913, rs763780	yes
CC	2	Lv	2015	Asian	264/264	rs2275913, rs763780	rs2275913 yes
		Quan	2012	Asian	311/463	rs2275913, rs763780	rs2275913 yes
HCC	2	Xi	2015	Asian	155/171	rs2275913, rs763780	no risk
		Li	2014	Asian	395/174	rs2275913	yes
PTC	1	Lee	2015	Asian	94/260	rs2275913	no risk
AML	1	Wrobel	2014	Caucasian	62/125	rs2275913, rs763780	rs763780 yes
EC	1	Yin	2014	Asian	380/380	rs2275913	yes
LC	1	Kaabachi	2014	Caucasian	239/258	rs2275913, rs763780	rs763780 yes
BLC	1	Zhou	2013	Asian	301/446	rs2275913, rs763780	yes
BC	1	Wang	2012	Asian	491/502	rs2275913, rs763780	rs2275913 yes
OC	1	Ruan	2012	Asian	92/38	rs2275913, rs763780	no risk

CRC: colorectal cancer; CC: cervical cancer; PTC: papillary thyroid cancer; HCC: hepatocellular carcinoma; LC: lung cancer; AML: acute myeloid leukemia; EC: esophageal cancer; BLC: bladder cancer; BC: breast cancer; OC: ovarian cancer.
